# Treatment of Pyorrhœa Alveolaris

**Published:** 1893-07

**Authors:** James Truman


### TREATMENT OF PYORRHŒA ALVEOLARIS.

BY JAMES TRUMAN, D.D.S.

In compliance with a desire often expressed, the following method, adopted by the writer, is given in as brief a manner as possible. It has no claim to originality, except in the use of two agents,—bicarbonate of soda and sulphate of quinine.

Abandoning all theories in regard to its etiology, the operator is asked to consider the state of the tooth and adjacent tissues. The tooth affected responds to a jar with a dull pain, indicating pericemental inflammation. The gum is loose and flabby. A probe indicates a pocket of varying depth. Calculi may possibly be found adherent to the cementum, not by any means a usual condition. Pus is manifested on pressure of the gum.

These being the representative diagnostic signs, the treatment resolves itself naturally into three distinct stages, which may arbitrarily be separated into primary, secondary, and permanent.

The *primary* is to remove all sources of irritation. This is accomplished, first, by the use of antiseptics and escharotics. (*a*) Hydrogen peroxide, followed by (*b*) mercuric chloride solution (1 to 2000). (*c*) Saturation of the pocket with commercial sulphuric acid, twenty-five-per-cent. solution. This is applied with a stick sharpened to the form of a chisel, carrying the acid down on all sides. The aromatic sulphuric acid is not adapted to this, as it fails in strength and contains undesirable ingredients. This is followed in a few moments by (*d*) an antacid, bicarbonate of soda preferred; making this into a magma with water and packing it in the pocket. The ebullition produced cleanses and also neutralizes all acid present.

The *secondary* is the filling of the pocket, on the conclusion of the primary treatment, with some agent which will prevent, for at least twenty-four hours, the development of pathogenic germs, and give time for natural processes in the formation of new tissue. This demands an antiseptic, readily formed into a paste and one not easily washed out. Very few agents are suitable for this. Aristol and quinine may be regarded as the best. The writer prefers the latter, for reasons not necessary to dilate upon here. The case is then left to be seen at a subsequent sitting,—the second or third day.

Upon the return of the patient the pocket is washed out with warm water and examined. If calculi be present, they are to be removed. Further treatment with any form of escharotic is not advisable. The quinine should be renewed, and the patient given the following mouth-wash, with instructions not to omit it a single day:

℞ Hydronaphthol, gr. xx;
Alcoholis,
Aquæ, āā ℥i. M.

Sig.—Half a teaspoonful of the above to a small tumblerful of water; to be used twice a day,—morning and before retiring at night.

The case can then be examined from time to time, but the antiseptic wash must be continued indefinitely. Upon this will depend the success of the treatment. Any omission will be certain to cause a relapse, as it is rarely possible to close entirely the pockets, and, unless they are kept aseptic, inflammation will be renewed. This latter constitutes the *permanent* treatment.

The philosophy of the method thus briefly outlined is based on the fact that, let the origin of this pathological condition be what it may, whether, as some assert, by systemic disturbances or, as others affirm, by purely local irritation, the incipient stages are marked, first, by inflammation at the gingival border, followed by rapid development of micro-organisms. It is evident that these pathogenic germs must be removed before any further effort be made.

This accomplished, other conditions may be considered. Calculus, if present, will be found to be a secondary product, adherent to the cementum, to be removed by scalers. This deposit holds, in the estimation of the writer, but a minor part in the irritation. The removal, combined with the antiseptic treatment, prepares the way for the use of a gentle escharotic. The object of this is to burn out any dead material present, giving the subjacent healthy tissues an opportunity to perform their normal function of restoration. This acid action should be only temporary, hence the use of an antacid to antagonize its possible destructive effects.

To accomplish a restoration of parts, protection from external influences must be secured. If this be not attended to, all the primary treatment becomes useless. This is at once the most difficult and uncertain, and, with ordinary remedies, generally results in failure. Hence the importance of employing an agent which will act as a protection to the pocket for at least twenty-four hours. It should be non-irritating and antiseptic, and be capable of being formed into a paste. This is found in the agent recommended,—quinine.

The investigations of Professor Binz, announced in 1867, demonstrated the fact that quinine, combined with human blood, at once arrests the diapedesis of the white blood-corpuscles. This has been "confirmed by other observers,—Scharrenbroich, Kerner, and Jerusalimsky." Binz also found that, when the mesentery of the living frog was placed under the microscope, quinine having been previously given, there was no aggregation of the white blood-cells in the vessels, nor did emigration occur upon irritation. "Dr. H. A. Hare (Professor H. C. Wood's 'Therapeutics') found that the vessels in the cinchonized frog were much more contracted and had the walls much thicker than in a corresponding frog without quinine. This contraction of the vessels is thought by Dr. Hare to be the result of a direct action exerted by the drug upon the muscular coat of the arterioles."

The somewhat remarkable dual character of quinine—a protoplasmic poison and antiseptic—made its use as a topical application a natural sequence. It was applied by myself some years ago as an experiment upon a fungoid presentation, so common between teeth with cavities on their proximal surfaces. Freely scarifying the parts to permit absorption of the agent, it was found that rapid resorption of the hypertrophied gum took place. This was further tested on the various forms of gingivitis with equally favorable results. It was then used in pyorrhœa alveolaris in the form of paste,—sulphate of quinine and water,—and with such satisfaction that it has become a standard therapeutic agent with myself in this disease.

The continued use subsequently of an antiseptic, in the form of a daily wash, is perhaps the most important of all, for, whatever treatment be used, if the patient fail to continue this, there will be a return of all the original symptoms. This will be understood when it is considered that the pocket rarely closes entirely, and any depression remaining invites to collection of the fluid contents of the mouth and subsequent fermentation.

The use of hydronaphthol is not only of great value in this lesion of the pericementum, but is also in all forms of gingivitis, and should be recommended for daily use in keeping rubber-plates free from irritating accretions. Where this or similar powerful antiseptics are used, of a non-toxic character, the inflammation of the mucous membrane, so commonly connected with the wearing of these plates, will, in most cases, be prevented. The plate should be kept at night in the solution, weakened by water. Thymol and creolin may be substituted for this; but, in the experience of the writer, this agent has proved effectual in all forms of oral inflammation, and in the amount named is non-irritating and free from any injurious effects.

